# The Late Professor Huxley

**Published:** 1895-08

**Authors:** 


### THE LATE PROFESSOR HUXLEY.

The death of Professor Thomas H. Huxley, June 29th, removes the last of that brilliant trio of English scientists, Darwin, Tyndall, and Huxley. Mr. Huxley was educated for a physician, receiving the doctor's degree from the University of London in 1845, with special honors in anatomy and physiology. After serving seven years in the English navy, he returned to London and was made professor of physiology and comparative anatomy in the University of London and in the Royal College of Surgeons. Outside of these studies Mr. Huxley said that he knew and cared little about medicine or the art of healing.

For the remainder of his life Mr. Huxley devoted all his energies to the study of biology and kindred sciences, and to the general popularization of science and to the dissemination of scientific knowlege. Among his most important works are "Man's Place in Nature," "Lay Sermons, Addresses and Reviews," "The Anatomy of Vertebrated Animals," "Origin of Species," "Critiques and Addresses," "Physical Basis of Life," etc., besides numerous able controversial papers in the magazines. In 1876 Mr. Huxley visited this country, delivering several lectures, which are published under the title "American Addresses." Mr. Huxley had no patience with that narrow spirit of the English church which set itself against the progress of scientific thought and investigation; and not the least achievement of his distinguished career was his lifelong and successful fight for the freedom of this thought and investigation against the blind dogmatism of a pharisaical clergy.

Mr. Huxley was in the highest sense a great man, and although an agnostic, he doubtless believed that there was "more faith in honest doubt than in half the creeds."

---

OUR esteemed neighbor, the *Southern Medical Record*, for several years edited and managed by Dr. D. H. Howell, is now being operated by Dr. Bernard Wolff. Dr. Wolff is eminently qualified for good literary and editorial work, and we take pleasure in wishing him success, and the *Record* a new lease of prosperity.

---

A GENTLEMAN in New Jersey had his appendix removed and found it to contain a large pearl, for which he was offered two hundred dollars. This announcement may still further stimulate the somewhat mad rush for appendices.

---

DR. J. B. S. HOLMES has resigned the Chair of Adjunct Professor of Obstetrics and Gynecology in the Southern Medical College, in order to devote his entire time to his private practice.

